# Social protection as a strategy for HIV prevention, education promotion and child marriage reduction among adolescents: a cross-sectional population-based study in Lesotho

**DOI:** 10.1186/s12889-024-18903-1

**Published:** 2024-06-06

**Authors:** Lucas Hertzog, Lucie Cluver, Boladé Hamed Banougnin, Maria Granvik Saminathen, Madison T. Little, Martina Mchenga, Rachel Yates, William Rudgard, Laura Chiang, Francis B. Annor, Viani Picchetti, Greta Massetti, Marisa Foraci, Rantsala Sanaha, Elona Toska

**Affiliations:** 1 Curtin School of Population Health, Faculty of Health Sciences, Curtin University, 400.233, Kent St, Bentley, Perth, WA 6102 Australia; 2 Centre for Social Science Research, University of Cape Town, Cape Town, South Africa; 3 WHO Collaborating Centre for Climate Change and Health Impact Assessment, Perth, Australia; 4 Department of Social Policy and Intervention, University of Oxford, Oxford, United Kingdom; 5 Department of Psychiatry and Mental Health, University of Cape Town, Cape Town, South Africa; 6 United Nations Population Fund, West and Central Africa Region Office, Dakar, Senegal; 7 Division of Violence Prevention, National Center for Injury Prevention and Control, US Centers for Disease Control and Prevention, Atlanta, USA; 8 UNICEF Lesotho, Maseru, Lesotho

**Keywords:** Social protection, Education, Sexual and reproductive health, HIV, Condom use, Multiple sexual partners, Transactional sex, Child marriage, Poverty, Adolescents, Sub-saharan Africa, Cash transfers

## Abstract

**Background:**

Lesotho’s government has shown consistent efforts to implement social protection programmes. However, while recent evidence established a positive causal relationship between some of these programmes and food security there is little evidence on the extent to which these initiatives are associated with better educational and sexual and reproductive health outcomes among vulnerable adolescents in Lesotho.

**Methods and Findings:**

The study uses cross-sectional, nationally representative data from the 2018 Lesotho Violence Against Children and Youth Survey. Our research examined the association between social protection receipt and educational and sexual and reproductive health outcomes among adolescents and young people (13–24 years) living in poverty. We employed multivariate logistic regression controlling for age, orphanhood, HIV status and sex. Social protection receipt was defined as household receipt of financial support from a governmental, non-governmental, or community-based program that provides income. Additionally, we fitted a marginal effects model by sex. Among the 3,506 adolescent females and males living in the two lowest poverty quintiles, receipt of social protection was associated with improvements in multiple adolescent outcomes: higher odds of consistent condom use (aOR 1.64, 95% CI 1.17–2.29), educational attainment (aOR 1.79, 95% CI 1.36–2.36), and school enrolment (aOR 2.19, 95% CI 1.44–3.34). Stratified analyses by sex showed that social protection receipt was also associated with reduced likelihood of child marriage among females (aOR 0.59, 95% CI 0.42–0.83) and higher odds of educational attainment and school enrolment among males (aOR 2.53, 95% CI 1.59–4.03 and aOR 3.11, 95% CI 1.56–6.19, respectively).

**Conclusions:**

Our study provides evidence that social protection programs are associated with improved educational, sexual and reproductive health and child marriage prevention outcomes among adolescents living in poverty. Implementing and expanding such social protection initiatives could prove instrumental in improving the well-being of vulnerable adolescents.

**Contributions:**

Social protection programs have been increasing in sub-Saharan African countries, playing a pivotal role in poverty reduction, with Lesotho being no exception. Despite the optimistic outlook brought about by the implementation of the National Social Protection Strategy Lesotho I (2014-19) and II (2021–2031), the impact of these programs on some specific outcomes that concern the lives of the most vulnerable adolescents in Lesotho remains to some extent unexplored. Additionally, Lesotho grapples with high rates of HIV, adolescent pregnancy, child marriage and early school dropout, which can further contribute to poor long-term health and social outcomes among adolescents. In this study, we used data from the 2018 Lesotho Violence Against Children and Youth Survey (VACS) to examine the association between receiving social protection and multiple adolescent outcomes: educational, sexual and reproductive. The findings revealed that social protection programs, particularly the existing government-provided cash transfers, are significantly associated with multiple better outcomes among adolescents living in the poorest households in Lesotho. Such cash transfer schemes in Lesotho are associated with improved sexual and reproductive health outcomes for adolescent females, including reduced child marriage rates, and improved educational outcomes for males. These findings indicate that government-led social protection programmes are positively associated with favourable outcomes that can improve the quality of life for adolescents in resource-limited settings.

**Supplementary Information:**

The online version contains supplementary material available at 10.1186/s12889-024-18903-1.

## Introduction

Since the mid-1990s, coverage of social protection programmes has been increasing in sub-Saharan African countries, playing a pivotal role in poverty reduction, yet more than 80% of the population remains unprotected [1–3]. However, this situation could change with an additional US$ 362.9 billion in investments annually in low- and middle-income countries. Such investments could profoundly impact the future of millions of young people, who represent a substantial and continuously growing demographic group in the coming decades. This demographic dividend has the potential to drive significant economic development for countries across the African continent [1, 4, 5].

In the past few decades, the Lesotho government has embraced the growing trend of introducing social protection strategies and schemes, including in collaboration with civil society with financial and technical support from a range of bilateral and multilateral institutions [6]. For example, Lesotho’s Ministry of Gender Youth Sport Art Culture and Social Development has improved an umbrella of different programmes with support from cooperating partners, such as the European Commission and UNICEF [7]. One notable program implemented at the national level and benefiting adolescents and children is the Child Grant, initiated in 2009, as an unconditional cash transfer scheme to reduce child malnutrition and improve child health and school enrolment, and to complement the important role already displayed by the Old Age Pensions introduced in 2004 to support orphaned and vulnerable children living in families affected by the HIV/AIDS epidemic [8]. In 2020, this program supported over 56,000 households, benefitting approximately 123,760 children and adolescents aged 0 to 17. These numbers are particularly remarkable for a country with historical challenges in achieving sustainable development. The progress has been made possible through a wide range of core and complementary social assistance and social security programmes with an annual budget of approximately US$ 120 million [7]. These programmes vary in eligibility criteria and benefit amount (Figure S3 and S4), and they may worth a significant proportion of the average household income in the country [9].

The National Social Protection Strategy Lesotho I (2014–2018) and II 2021–2031 were formulated with the support of UNICEF in collaboration with various institutions such as the World Food Programme (WFP), the Food and Agriculture Organization (FAO), and the World Bank together with a multitude of regional and local NGOs [7]. Although both strategies recognise the importance of social protection, the impact of the existing programmes on the lives of adolescents most susceptible to poverty in Lesotho has not been explored. Existing evidence suggests that social protection has positively influenced the lives of hundreds of thousands of people in the country [10–15]. Still, economic deprivation continues to be widespread in Lesotho, with estimates indicating that while around 50% of the population lives in monetary poverty and 19.6% in multidimensional poverty, more than 75% of the population is either poor or susceptible to poverty [16]. While these headcounts are staggering at the household level it is also worth noting that as of 2018 45.5% of children aged 0 to 17 and 57.9% of Basotho children aged 13 to 17 years lived in multidimensional poverty [17].

These statistics serve as a poignant reminder of the significant challenges the vast portion of the population, especially adolescents, face, which put them at higher risk of unfavourable outcomes in various aspects of their lives. A robust and consolidated body of studies demonstrates that poverty can undermine crucial outcomes such as quality education and health and their mutual reinforcement on adolescent well-being [18–22]. Lesotho additionally contends with high rates of HIV prevalence and adolescent pregnancy, further compounding poor long-term outcomes among adolescents [23]. Studies have highlighted the negative impacts of HIV, adolescent pregnancy, and child marriage on adolescent health, including increased vulnerability to other health issues and decreased educational opportunities [24]. It is thus crucial to explore the impact of a promising and evolving social protection system in the lives of individuals who are simultaneously most vulnerable and hold an integral, transformational capacity in shaping the region’s future.

The 2018 Lesotho Violence Against Children and Youth Survey (VACS) provides a unique opportunity to examine these associations. It is a comprehensive, nationally representative study conducted among adolescents and young people in Lesotho, providing valuable insights into adolescent health and well-being, including sexual and reproductive health and educational outcomes [25–28].

Our study capitalises on VACS data collected during the implementation of the National Social Protection Strategy Lesotho I 2014–2018 and scale-up of child support grants, which creates an opportunity to improve our understanding of how existing programmes impact the lives of vulnerable adolescents. Standardised questionnaires were implemented among a nationally representative sample, strengthening generalisability and comparability with similar lower-middle-income country (LMIC) contexts [29, 30]. Accordingly, this study hypothesise that social protection receipt in a household is associated with reduced adolescent risk exposure, and has three aims: (i) to determine the association between receipt of social protection and multiple educational, and sexual and reproductive health outcomes, (ii) to assess the differential associations between social protection and adolescent outcomes, and (iii) to test associations between governmental and non-governmental social protection schemes and potential benefits for HIV prevention and education promotion among adolescents.

## Methods

### Study design, sampling and procedures

The 2018 Lesotho VACS was a nationally representative cross-sectional household survey aimed at assessing the prevalence of adolescent and young people’s experiences of violence across the country. The national geopolitical subdivisions from the 2016 census compiled by the Lesotho Bureau of Statistics served as the basis for the sampling frame. The survey collected data from males and females aged 13–24, considered as adolescents and young people [31]. A separate, short survey was conducted with the household head. The survey used a three-stage sample design. In the first stage, 240 primary sampling units (PSUs) were selected using probability proportional to size (43 male PSUs and 197 female PSUs were selected - females had many more PSUs because females were over-sampled due to the implementation funder’s interest in collecting more granular data on adolescent girls and young women, a priority population with disproportionately higher risk for HIV). In the second stage, from each of the 240 PSUs, 40 households were randomly selected. In the third and final stage, one eligible individual aged 13–24 from each household was selected depending on whether it was a male or female PSU. Lesotho VACS used a split sample approach, interviewing females and males in different communities (resulting in Female PSUs and Male PSUs) to safeguard the confidentiality of participants by reducing the chance that both a perpetrator and a victim from different sexes would be interviewed [30]. Field pre-testing was conducted before data collection. Interviews were conducted in safe and secure locations to ensure confidentiality and encourage disclosure using a structured questionnaire. Answers were recorded on netbook computers using the CSPro platform. Interviews with heads of household were15-minute assessments to collect socio-economic information about the household.

From June to September 2018, 7,101 females and 1,467 males completed in-person interviews in English or Sesotho. Overall response rate was 96.2% each for females and males. After completion of the questionnaire, participants who did not report a previous positive HIV test were offered HIV testing with pre-and post-test counselling following national and international guidelines [32, 33]. Individuals who were cognitively impaired or living with a physical disability (such as those with severe hearing or speech impairment) were ineligible to participate in the survey.

### Measures

The independent variables were measured through two questions that were asked to the head of each selected household, namely if anyone received outside financial help from (a) a governmental program (Does anyone in the household receive outside financial help from a government program?) and/or (b) a non-governmental program or participated in a community-based program that provides income, such as microfinance, loan, or community savings group (Does anyone in the household receive outside financial help from a non-government program, or does someone participate in a community based program that provides income, such as micro finance, loan, or community savings group?). Dependent variables measured were sexual and reproductive health outcomes (consistent condom use, multiple sexual partners, and transactional sex), child marriage and educational-related outcomes (schooling and paid work activities), and questions were asked to the selected 13–24-year-old respondent. Consistent condom use was measured by self-reported always having used condoms with the last three sexual partners in the previous 12 months among those who ever had sex. Child marriage was assessed by asking the participant’s ages when they first got married or started living together as if married, among those who had indicated they had been married. Responses indicating marriage at less than 18 years old were defined as child marriage [34]. Participants who reported having had more than one sexual partner in the previous 12 months were defined as having multiple sexual partners. Transactional sex in the past 12 months was defined as responding yes to a question about ever having sex with any of the last three sexual partners (in the previous 12 months, among those who had already had sex) mainly to get things they needed, such as money, gifts, or other things that were important to participants. Educational variables measured educational attainment (currently attending or completed primary school or lower; currently attending or completed any education higher than primary school - depending on the participants’ age according to Lesotho’s educational system) and whether a participant was currently enrolled in school (attending school; not attending school). Engagement in any paid work was defined as engaging in any paid work as an employee or self-employed individual in the previous 12 months of the interview, a question only asked for participants over 18 years old. The independent and dependent variables were coded as binary indicators for analysis. The analysis controlled for age, sex (in the non-stratified analyses), orphanhood (having lost one or both parents) status, and HIV status. Other controls were tested in initial analyses but not included in the final model as have not improved the explanatory power. Covariates were categorical variables, except for participants’ age, which was continuous.

### Analysis

Data were prepared in Stata 16.1, and analyses were conducted in R using a reproducible pipeline with the *targets* package to facilitate complex workflow tasks and control for objects’ dependencies (Figure S1). Data preparation involved recoding independent, dependent, and control variables and sample selection of the adolescents (age groups 13 to 17 and 18 to 24) most susceptible to poverty (lower two wealth quintiles). Data analysis was conducted in nine stages. First, a Principal Component Analysis (PCA) was conducted, including items used in large surveys concerning standards of living as a wealth index [35] (water supply, type of toilet, cooking fuel, house floor, roof, and wall materials, asset ownership including livestock, herds, and other farm animals, and number of persons per room in the household – questions included in the VACS head of household questionnaire). Given that all these variables are binary categorical (0 or 1), they were standardised using the *means* option in Stata. In this context, the mean of a binary variable represents the proportion of observations with a value of 1. The standardisation process centres each variable around this proportion and scales it by its standard deviation. This approach ensures that each variable contributes to the PCA based on its relative variability within the dataset, facilitating a balanced representation of the derived components. Second, wealth index scores were predicted for each respondent based on the results of the PCA. Third, a quintile categorisation was performed following an approach used in studies in similar settings [36, 37] (first and second quintiles considered very poor; upper quintiles less poor), and a subsample of 3,506 (the two poorest quintiles) was selected for analysis given the characteristics of Lesotho’s pro-poor social protection strategy [7]. Fourth, a function-oriented reproducible pipeline was constructed in R after variable and subsample selection (Figure S1). Fifth, survey design was declared to account for the three-stage sampling and ensure all analyses yielded nationally representative results, and sub-samples were selected for sex stratification. Sixth, we conducted descriptive analyses stratified by sex and compared potential differences between sexes using Wilcoxon rank-sum test for complex survey samples (continuous variable) and chi-squared tests with Rao & Scott’s second-order correction (categorical variables). Seventh, associations between exposure and outcomes were examined using multivariate logistic regressions. Adjusted odds ratios (aORs) with 95% confidence intervals (CIs) were calculated to assess the strength of the associations, controlling for sociodemographic characteristics. Missing data were less than 4% (Table S4) and missingness was handled using listwise deletion. Eighth, estimated *p*-values were adjusted using the Benjamini-Hochberg procedure specified with a false discovery rate of 5% to account for the risk of type I error from multiple-hypothesis testing [38]. Finally, predicted percentage probabilities were calculated using marginal effects modelling for outcomes with significant associations using the *ggeffects* package. Adjusted predicted percentage probabilities for experiencing outcomes when adolescent and young people households reported no social protection provision compared to social protection provision receipt were calculated for each outcome of interest. Differences in percentage probability were examined to assess the impact of social protection receipt (government and non-government programmes) on the outcomes, and absolute and relative differences were estimated.

## Results

### Descriptive statistics

**Fig. 1:**
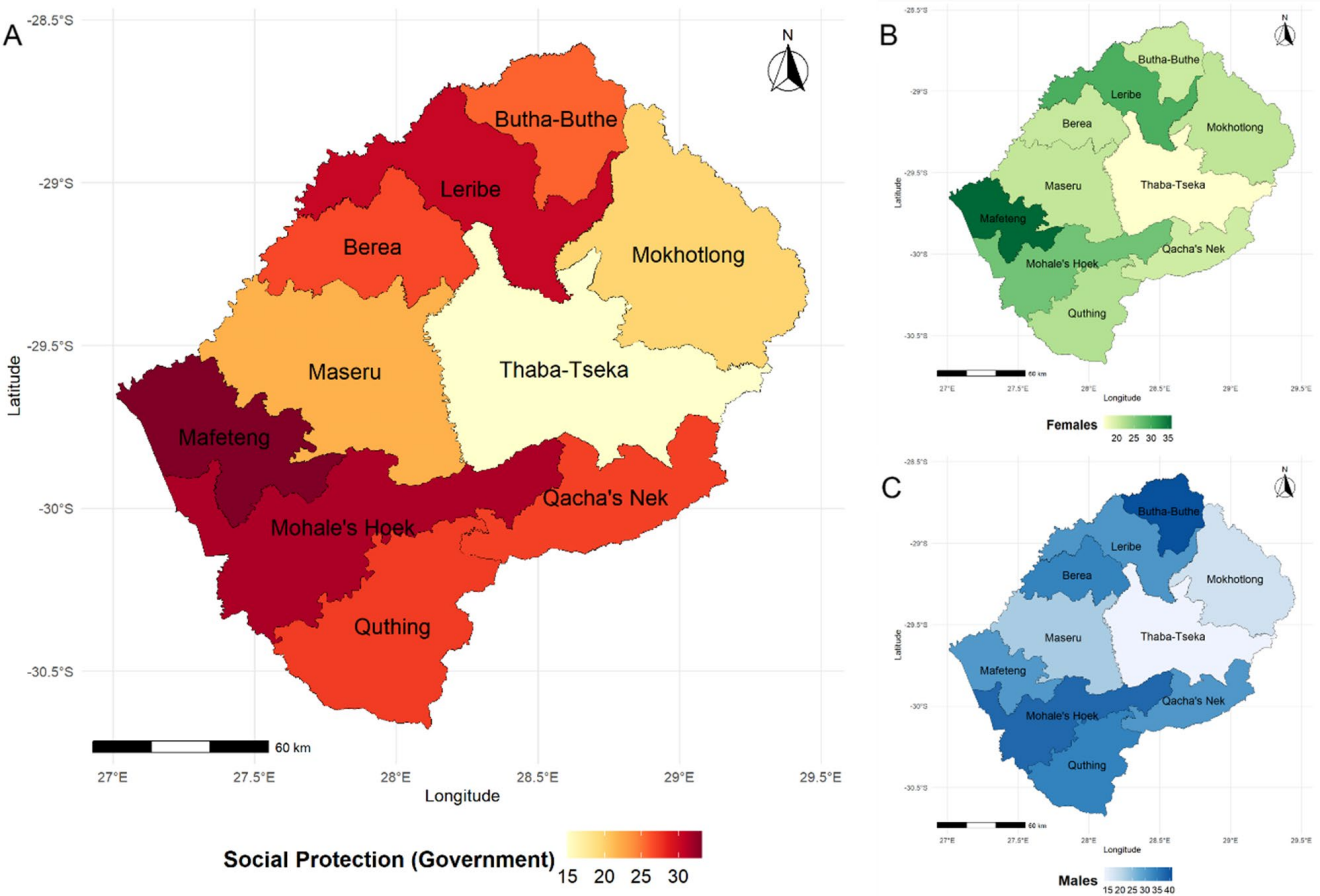
Summary of weighted % of households receiving governmental social protection by district in Lesotho. **A**. Whole sample. **B**. Percentage of Females by district who live in households covered by social protection. **C**. Percentage of Males by district who live in households covered by social protection

The study sample consisted of 3,506 adolescents and young people aged 13–24 years living in poverty (poorest 40% households) in Lesotho, with a mean age of 18 years. Among the study subsample, 48% had lost at least one of their biological parents, and 4% were living with HIV (with a significantly higher HIV prevalence among adolescent females). Frequency distributions for sociodemographic characteristics, the prevalence of social protection provision, and educational and sexual and reproductive health outcomes are shown in Table 1 (also see Figure S2). Among youth living in poverty aged 13–24 years, 25% received social protection through governmental and 6.3% from non-governmental programmes. Figure 1 gives an overview of governmental programmes’ distribution across districts. Almost half of the adolescents were enrolled in school (47%), and a similar percentage was observed in educational attainment (completed higher than primary school). Adolescent females had relatively better educational outcomes, with 56% having completed higher than primary school compared to 37% of males (*p* < 0.001). Even though males had more protected sex than females, 68–37% (*p* < 0.001), they were more likely to have multiple sexual partners (28–7.2%, *p* < 0.001). Females faced significantly higher risks of being married before 18 years (13–0.8%, *p* < 0.001).

**Table 1:** Sociodemographic characteristics of 13-24-year-old adolescents and young people living in poverty (lower two wealth quintiles of the total VACS Lesotho sample)

Characteristic	Total (*N* = 3,506)^1^	Males (*N* = 625)^1^	Females (*N* = 2,881)^1^	*p*-Value^2^
Age - mean (SD)	18.0 (3.4)	18.0 (3.4)	17.9 (3.4)	0.5^3^
Age Groups				0.3^4^
13–17	46%	45% (40 – 49%)	47% (45 – 50%)	
18–24	54%	55% (51 – 60%)	53% (50 – 55%)	
Living with HIV	4.1%	2.5% (1.2 – 5.0%)	5.8% (4.7 – 7.2%)	0.018* ^4^
Orphanhood	48%	49% (44 – 55%)	47% (45 – 50%)	0.5^4^
**Provision**				
Social protection (Non-Govt.)	6.3%	8.1% (4.4 – 14%)	4.5% (3.5 – 5.8%)	0.073^4^
Social protection (Govt.)	25%	27% (23 – 32%)	23% (20 – 26%)	0.2^4^
**Outcomes**				
Enrolled in school	47%	44% (37 – 50%)	50% (46 – 53%)	0.11^4^
Educational attainment (Completed higher than primary school)	46%	37% (29 – 46%)	56% (52 – 60%)	< 0.001*** ^4^
Engaged in any paid work (over 18 yrs)	16%	22% (18 – 26%)	11% (9.5 – 14%)	< 0.001*** ^4^
Consistent condom use	53%	68% (60 – 76%)	37% (34 – 40%)	< 0.001*** ^4^
Multiple sexual partners	18%	28% (20 – 37%)	7.2% (5.6 – 9.1%)	< 0.001*** ^4^
Transactional sex	3.2%	1.9% (0.59 – 5.8%)	4.6% (3.2 – 6.6%)	0.12^4^
Child marriage	6.8%	0.8% (0.32 – 1.9%)	13% (11 – 15%)	< 0.001*** ^4^

SD = Standard Deviation; CI = confidence interval; Bold values indicate significance at *p* < 0.05.

^1^ Weighted % (95% CI].

^2^ *p < 0.05; **p < 0.01; ***p < 0.001.

^3^ Wilcoxon rank-sum test for complex survey samples.

^4^ chi-squared test with Rao & Scott’s second-order correction.

### Associations between social protection and outcomes

#### Government social protection

**Fig. 2:**
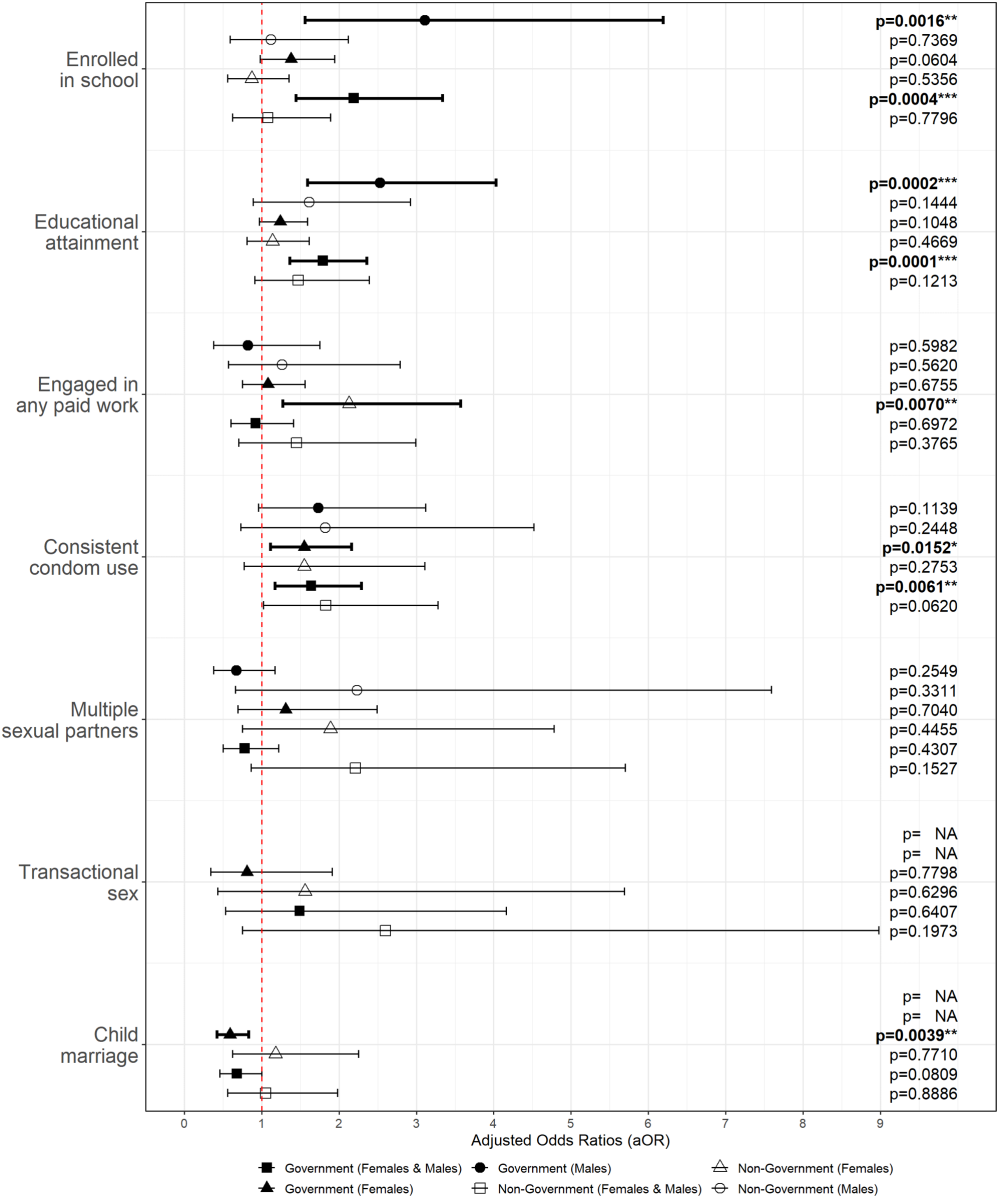
Forest plot with multivariable associations results by social protection programme source and sex. *Engagement in any paid work only refers to participants over 18 years old

Receipt of Governmental social protection programmes was associated with both males’ and females’ educational and reproductive health outcomes (Table 2; Fig. 2). Receipt of social protection through governmental programmes was significantly associated with higher odds of enrolment in school for both males and females. Adolescents in households covered by government programmes were more than twice as likely to be enrolled in school with adjusted odds ratio (aOR) = 2.19 (95% CI 1.44 to 3.34, *p* < 0.0001).

**Table 2:** Summary of multivariable associations between provisions and outcomes

		Social protection (Non-Govt.)		Social protection (Govt.)	
**Females & Males** ^**5**^	N^1^	aOR (95% CI)	*p*-Value^2^	aOR (95% CI)	*p*-Value^2^
Enrolled in school	3,305	1.08 (0.62–1.89)	0.7796	**2.19 (1.44–3.34)**	0.0004***
Educational attainment^3^	3,374	1.47 (0.91–2.39)	0.1213	**1.79 (1.36–2.36)**	0.0001***
Engaged in any paid work^4^	3,281	1.45 (0.70–2.99)	0.3765	0.92 (0.60–1.41)	0.6972
Consistent Condom Use	1,654	1.83 (1.02–3.28)	0.0620	**1.64 (1.17–2.29)**	0.0061**
Multiple Sexual Partners	1,659	2.21 (0.86–5.70)	0.1527	0.78 (0.50–1.22)	0.4307
Transactional Sex	1,465	2.6 (0.75–8.98)	0.1973	1.49 (0.53–4.16)	0.6407
Child Marriage	3,371	1.05 (0.56–1.98)	0.8886	0.68 (0.46–1.00)	0.0809
**Females**					
Enrolled in school	2,723	0.87 (0.56–1.35)	0.5356	1.38 (0.98–1.94)	0.0604
Educational attainment^3^	2,764	1.14 (0.81–1.61)	0.4669	1.24 (0.97–1.59)	0.1048
Engaged in any paid work^4^	2,703	**2.13 (1.27–3.57)**	0.0070**	1.08 (0.75–1.56)	0.6755
Consistent Condom Use	1,334	1.55 (0.77–3.11)	0.2753	**1.55 (1.11–2.16)**	0.0152*
Multiple Sexual Partners	1,339	1.89 (0.75–4.78)	0.4455	1.31 (0.69–2.49)	0.7040
Transactional Sex	1,204	1.56 (0.43–5.69)	0.6296	0.81 (0.34–1.91)	0.7798
Child Marriage	2,761	1.18 (0.62–2.25)	0.7710	**0.59 (0.42–0.83)**	0.0039**
**Males**					
Enrolled in school	582	1.12 (0.59–2.12)	0.7369	**3.11 (1.56–6.19)**	0.0016**
Educational attainment^3^	610	1.61 (0.89–2.92)	0.1444	**2.53 (1.59–4.03)**	0.0002***
Engaged in any paid work^4^	578	1.26 (0.57–2.79)	0.5620	0.82 (0.38–1.75)	0.5982
Consistent Condom Use	320	1.82 (0.73–4.52)	0.2448	1.73 (0.96–3.12)	0.1139
Multiple Sexual Partners	320	2.23 (0.66–7.59)	0.3311	0.67 (0.38–1.17)	0.2549
Transactional Sex		†		†	
Child Marriage		†		†	

aOR = adjusted odds ratio; CI = confidence interval; Bold values indicate significance at *p* < 0.05.

^1^ Sample size differences reflect the survey characteristics using gateway questions.

^2^ *p < 0.05; **p < 0.01; ***p < 0.001.

^3^ Completed higher than primary school.

^4^ Participants over 18 years old.

^5^ The analysis controlled for age, sex (in the non-stratified analyses), orphanhood (having lost one or both parents) status, and HIV status.

† Result suppressed as the estimate is unreliable due to small cell size.

Regarding sexual and reproductive health outcomes, findings showed that females in households receiving governmental social protection benefits were more likely to use condoms consistently. Additionally, females in households covered by social protection programs had lower odds of marrying before 18.

The significance of governmental social protection coverage also extended to males’ education. Males living in households covered by government-led social protection programmes had significantly higher odds of being enrolled in school (aOR of 3.11, 95% CI 1.56 to 6.19, *p <* 0.01) and achieving better educational attainment (aOR of 2.53, 95% CI 1.59 to 4.03, *p* < 0.001). It should be noted that due to small cell sizes and unreliability, the results for transactional sex and child marriage among males were suppressed, highlighting the need for further investigation in these areas.

#### Non-government social protection

Non-governmental social protection programs did not show any significant associations with positive adolescent outcomes when considering the entire sample. However, an important finding emerged when examining girls and young women over 18 years old (sub-sample who was asked if engaged in any paid work as an employee or self-employed, not asked for those under 18). In this subgroup, non-governmental social protection programs demonstrated a significant association with engagement in paid work (aOR = 2.13, 95% CI 1.27 to 3.57, *p <* 0.01).

### Outcomes probabilities

Table 3 presents the adjusted predicted percentage probabilities for experiencing outcomes considering social protection receipt in the household, highlighting the differences in absolute and relative terms. For both females and males, the predicted percentage probability of being enrolled in school was higher among those who received social protection compared to those without coverage, with similar differences in percentage (ranging from 11.2 to 18.7% in absolute terms) for other outcomes such as educational attainment and consistent condom use.

Females over 18 and in households covered by non-governmental programmes doubled their chances of engaging in paid work compared to females living in uncovered households. The receipt of governmental financial support was associated with higher predicted percentage probabilities of consistently using condoms in the previous 12 months (10.3% difference in absolute terms) and was associated with substantial reductions in probabilities of being married before 18 years (-37.9% difference in relative terms).

Males in households with social protection coverage had substantially higher predicted percentage probabilities of being enrolled in school and achieving higher educational attainment compared to those without social protection. The absolute differences were 27.1% (relative difference of 91.2%) for enrolment in school and 19.7% (relative difference of 88.7%) for educational attainment.

**Table 3:** Adjusted predicted percentage probabilities for experiencing outcomes and no social protection compared to social protection receipt

			Difference in % probability compared to no social protection	
**Females & Males**	**No social protection** ^**1**^	**Social protection** ^**1**^	Absolute	Relative
Enrolled in school	49.6 (43.4–55.8)	68.3 (59–76.4)	18.7	37.7
Educational attainment^2^	57.5 (51.9–62.8)	70.8 (63.1–77.4)	13.3	23.1
Consistent Condom Use	30 (24.6–36)	41.2 (33.9–48.9)	11.2	37.3
**Females**				
Engaged in any paid work^3^	4.6 (3.3–6.2)	9.2 (5.4–15.3)	4.6	100.0
Consistent Condom Use	33.6 (28.8–38.7)	43.9 (36.3–51.8)	10.3	30.7
Child Marriage	13.2 (11–15.9)	8.2 (5.5–12.2)	-5.0	-37.9
**Males**				
Enrolled in school	29.7 (18.4–44.1)	56.8 (45.5–67.4)	27.1	91.2
Educational attainment^2^	22.2 (15.2–31.3)	41.9 (31.1–53.7)	19.7	88.7

^1^ Values are expressed in percentages with lower and upper bounds of the confidence interval in parentheses.

^2^ Completed higher than primary school.

^3^ Non-governmental social protection programme, only participants over 18 years old.

## Discussion

In this study, we have analysed associations of social protection programs on educational and sexual and reproductive health outcomes among adolescents and young people living in poverty in Lesotho, emphasising the potential associations with government-led and non-government-led programmes. The findings reinforce the role of national governments in promoting protective measures for their population, in line with the Lesotho National Social Protection Strategy II vision of reducing economic and social vulnerabilities and alleviating poverty and deprivation.

A central result of our research complements findings from other countries, illustrating how social protection interventions can significantly improve sexual reproductive health and educational outcomes among adolescents [39–41]. Examining sexual and reproductive health, the disproportionate impact of HIV on girls found in Lesotho and other resource-limited settings [42] was also evident in our study. This pattern has been attributed to socio-historical contingencies detrimental to women, such as gender-based violence and inequalities [43–45]. Our findings lend further urgency to initiatives that aim to reduce HIV incidence among girls and young women in low-income settings; the promising association between social protection receipt in the household and girls’ consistent condom use suggest that access to government-induced economic strengthening strategies is associated with safer sexual behaviour.

Government social protection receipt was significantly positively associated with consistent condom use for the overall sample and females separately, but this association was not observed for males, which may reflect the differences in sample sizes (larger for females). Potential reasons for this discrepancy warrant further investigation.

Our analysis also provides compelling evidence of a significant positive association between social protection coverage and school enrolment and educational attainment, respectively. These findings not only support but also extend the conclusions from previous studies on the impact of social protection on outcomes among Lesotho’s youth [46] and their peers in other comparable contexts [3]. However, while males exposed to social protection had higher odds of educational attainment than those living in households not covered by programmes, our study did not find any significant effects on educational attainment for females.

Meanwhile, lower odds of risky sexual behaviour and an important association with lower rates of child marriage were observed among adolescent females. Considering that adolescence is a critical stage for establishing lifelong sexual and reproductive health behaviours, the potential of social protection programs to influence these outcomes is key. This underscores the complex interplay between social determinants, gender norms, and population health outcomes and calls for the implementation and scale-up of gender-sensitive social protection programs that align with the objectives of many international health frameworks [16–20].

While the positive outcomes of social protection programmes were more evident with governmental initiatives, non-governmental programs may have played a vital role for females over 18 years, being positively associated with young women’s engagement in paid work. This suggests the potential of these programs to foster economic empowerment and provide opportunities for young women despite potential challenges triggered by economic empowerment, such as intimate partner violence victimisation due to earning more than their partners [47].

Our findings also highlight a high incidence of orphanhood among adolescents in poverty, a legacy of the HIV epidemic and an issue of considerable concern in low-income settings due to its association with higher vulnerability to poverty, abuse, and exploitation, and with increased risks as a consequence of the COVID-19 pandemic [48]. These findings underline the need for social protection mechanisms sensitive to the additional layers of vulnerability brought by the loss of parents and caregivers.

While providing a valuable contribution to the existing literature about the impact of social protection on adolescent outcomes in resource-limited settings, our study has limitations. Its cross-sectional design restricts the capacity to draw temporal and causal conclusions from the observed associations, which might affect interpretations regarding the impacts of social protection programmes. Potential biases, including selection bias and information bias, must be acknowledged as the study relied on self-reported data, which may be influenced by recall or social desirability bias. These biases may be particularly important in our context as adolescents report on sensitive issues, potentially leading to over- or under-reporting. Moreover, despite adjustments for several potential confounders, the possibility of residual confounding due to unmeasured or imperfectly measured factors remains. Missing data was low overall (less than 4%) but might still introduce bias by under-or overestimating values. In addition, the VACS questionnaires do not specify which specific social protection programme is being received at the household level, presenting challenges in interpreting the effectiveness of distinct strategies tailored for adolescents and differentiating between diverse governmental and non-governmental programs. Such programs, whether governmental or non-governmental, could substantially vary in their design, implementation, and impact across regions or families. Hence, comparisons between programmes are limited and should be interpreted with caution and the different sample sizes for females and males may also be noted.

Notwithstanding these limitations, our study contributes to understanding the granularity of how investments focused on vulnerable populations may have multiplier effects in the household, with benefits encompassing families and communities beyond the recipient. Future research could benefit from building upon our findings by investigating in greater depth the differences in effects between government-led and non-government-led programmes, the differences between promotive, preventive and transformative initiatives, and exploring the reasons behind these differences. Further, it would be valuable to explore ways of enhancing the effectiveness of initiatives that specifically respond to the needs of adolescents and social groups with intersecting vulnerabilities.

Finally, our study provides evidence that social protection receipt is associated with improved well-being for adolescents living in poverty in Lesotho, considering outcomes measured. The findings suggest that social protection programmes may promote safe sex practices and learning opportunities that may lead to economic stability and empower adolescents. Such improvements in adolescent outcomes are likely to have multiplier effects as they transition into adulthood for adolescents, their children and their families [49]. The National Social Protection Strategy Lesotho II is a vital step to accelerate progress through which adolescents and young people in Lesotho may reframe their contribution to society. Ensuring that its implementation reaches the poorest households with the most vulnerable adolescents is critical to maximising its impact. Our findings confirm the potential of targeted social protection programmes in Lesotho and similar settings and highlight the potential benefits associated with their expansion and capillarity to reach more adolescents.

### Electronic supplementary material

Below is the link to the electronic supplementary material.

Supplementary Material 1

## Data Availability

Data is available upon request on https://www.togetherforgirls.org/en. The code in R used for analysis is available on https://github.com/lucashertzog/ResPrj_VACS_Lesotho_2018_Social_Protection.
