# Supplementary material for: Social protection as a strategy for HIV prevention, education promotion and child marriage reduction among adolescents: a cross-sectional population-based study in Lesotho
Source: BMC Public Health. 2024 Jun 6;24:1523. doi: 10.1186/s12889-024-18903-1 (PMC11157706; doi:10.1186/s12889-024-18903-1)
Supplement: Supplementary file 1 — Supplementary Material 1 [file 12889_2024_18903_MOESM1_ESM.docx]

Supplementary material for

Social protection as a strategy for HIV prevention, education promotion and child marriage reduction among adolescents: a cross-sectional population-based study in Lesotho

Lucas Hertzog*^1,2,3^, Lucie Cluver*^4,5^, Boladé Hamed Banougnin^2,6^, Maria Granvik Saminathen^2^, Madison T. Little^4^, Martina Mchenga^2^, Rachel Yates^4^, William Rudgard^4^, Laura Chiang^7^, Francis B. Annor^7^, Viani Picchetti^7^, Greta Massetti^7^, Marisa Foraci^8^, Rantsala Sanaha^8^, Elona Toska^2,4^.

* Joint first authors

1. Curtin School of Population Health, Faculty of Health Sciences, Curtin University, Perth, Australia.

2. Centre for Social Science Research, University of Cape Town, Cape Town, South Africa.

3. WHO Collaborating Centre for Climate Change and Health Impact Assessment, Perth, Australia.

4. Department of Social Policy and Intervention, University of Oxford, Oxford, United Kingdom.

5. Department of Psychiatry and Mental Health, University of Cape Town, Cape Town, South Africa.

6. United Nations Population Fund, West and Central Africa Region Office, Dakar, Senegal.

7. Division of Violence Prevention, National Center for Injury Prevention and Control, US Centers for Disease Control and Prevention, Atlanta, USA.

8. UNICEF Lesotho, Maseru, Lesotho.

# S1 Appendix

Table S1. Correlations between outcomes.

| **Variable** | **1** | **2** | **3** | **4** | **5** | **6** |
| --- | --- | --- | --- | --- | --- | --- |
| 1. Enrolled in school |  |  |  |  |  |  |
| 2. Educational attainment (completed higher than primary school) | .07**  (.04, .10) |  |  |  |  |  |
| 3. Engaged in any paid work (over 18 yrs) | -.34**  (-.37, -.31) | .00  (-.03, .04) |  |  |  |  |
| 4. Consistent Condom Use | .29**  (.25, .33) | .10**  (.05, .15) | .04  (-.01, .08) |  |  |  |
| 5. Multiple Sexual Partners | .05*  (.00, .10) | .02  (-.02, .07) | .04  (-.01, .09) | .01  (-.04, .05) |  |  |
| 6. Transactional Sex | -.02  (-.07, .03) | -.03  (-.08, .02) | .03  (-.02, .08) | .02  (-.03, .07) | .15**  (.10, .20) |  |
| 7. Child Marriage | -.31**  (-.34, -.28) | -.07**  (-.11, -.04) | .01  (-.02, .05) | -.25**  (-.30, -.21) | -.09**  (-.14, -.05) | -.01  (-.06, .04) |
| Values in parentheses indicate the 95% confidence interval for each correlation. | | | | | | |
| * *p* < 0.05. **; *p* < 0.01. | | | | | | |

Table S2. Sociodemographic characteristics of 13-24-year-old adolescents and young people living in poverty (lower two wealth quintiles of the total VACS Lesotho sample) by receipt of governmental social protection programme in the household.

|  | **Social Protection Receipt (Government)** | | | |
| --- | --- | --- | --- | --- |
|  | **No** | **Yes** | **No** | **Yes** |
| **Characteristic** | **Males (N=447)**^1^ | **Males (N=166)**^1^ | **Females (N=2,136)**^1^ | **Females (N=648)**^1^ |
| Age – mean (SD) | 18.2 (3.5) | 17.4 (3.0) | 18.0 (3.4) | 17.3 (3.3) |
| Age Groups |  |  |  |  |
| 13-17 | 43% [38%, 48%] | 50% [40%, 61%] | 45% [42%, 48%] | 57% [53%, 61%] |
| 18-24 | 57% [52%, 62%] | 50% [39%, 60%] | 55% [52%, 58%] | 43% [39%, 47%] |
| Living with HIV | 2.3% [0.82%, 6.2%] | 3.2% [1.1%, 8.7%] | 6.4% [5.0%, 8.0%] | 4.2% [2.8%, 6.2%] |
| Orphanhood | 47% [40%, 54%] | 57% [49%, 64%] | 44% [41%, 47%] | 55% [50%, 60%] |
| Enrolled in school | 37% [30%, 44%] | 57% [48%, 67%] | 46% [42%, 50%] | 57% [53%, 62%] |
| Educational attainment^2^ | 32% [24%, 40%] | 47% [36%, 58%] | 54% [50%, 58%] | 57% [50%, 63%] |
| Engaged in any paid work^3^ | 24% [19%, 30%] | 16% [10%, 25%] | 11% [9.0%, 13%] | 9.1% [7.0%, 12%] |
| Consistent condom use | 66% [56%, 74%] | 78% [65%, 86%] | 34% [30%, 38%] | 46% [39%, 53%] |
| Multiple sexual partners | 28% [21%, 38%] | 19% [12%, 31%] | 6.9% [5.2%, 9.2%] | 8.8% [5.4%, 14%] |
| Transactional sex | 1.3% [0.18%, 8.2%] | 4.4% [1.2%, 15%] | 4.9% [3.3%, 7.4%] | 4.2% [2.2%, 7.8%] |
| Child marriage | 0.6% [0.16%, 2.1%] | 1.4% [0.36%, 5.4%] | 15% [13%, 17%] | 8.7% [6.2%, 12%] |

SD = Standard Deviation; CI = confidence interval.

^1^ Weighted % (95% CI).
^2^ Completed higher than primary school.

^3^ Participants over 18 years old.

Table S3. Sociodemographic characteristics of 13-24-year-old adolescents and young people living in poverty (lower two wealth quintiles of the total VACS Lesotho sample) by receipt of non-governmental social protection programme in the household

|  | **Social Protection Receipt (Non-Government)** | | | |
| --- | --- | --- | --- | --- |
|  | **No** | **Yes** | **No** | **Yes** |
| **Characteristic** | **Males (N=569)**^1^ | **Males (N=44)**^1^ | **Females (N=2,641)**^1^ | **Females (N=141)**^1^ |
| Age – mean (SD) | 18.1 (3.4) | 17.4 (3.0) | 17.9 (3.4) | 17.4 (3.1) |
| Age Groups |  |  |  |  |
| 13-17 | 44% [40%, 49%] | 52% [37%, 66%] | 48% [45%, 50%] | 54% [44%, 63%] |
| 18-24 | 56% [51%, 60%] | 48% [34%, 63%] | 52% [50%, 55%] | 46% [37%, 56%] |
| Living with HIV | 2.6% [1.2%, 5.5%] | 1.8% [0.22%, 13%] | 5.9% [4.7%, 7.4%] | 4.6% [2.5%, 8.3%] |
| Orphanhood | 52% [45%, 58%] | 30% [15%, 52%] | 47% [44%, 49%] | 50% [40%, 60%] |
| Enrolled in school | 42% [36%, 48%] | 50% [33%, 68%] | 48% [45%, 52%] | 51% [43%, 58%] |
| Educational attainment^2^ | 35% [27%, 43%] | 46% [29%, 64%] | 55% [50%, 59%] | 57% [47%, 66%] |
| Engaged in any paid work^3^ | 22% [18%, 26%] | 18% [8.3%, 35%] | 10% [8.7%, 12%] | 15% [9.4%, 22%] |
| Consistent condom use | 68% [59%, 75%] | 83% [67%, 92%] | 36% [32%, 40%] | 47% [32%, 63%] |
| Multiple sexual partners | 25% [18%, 34%] | 42% [20%, 69%] | 7.1% [5.5%, 9.2%] | 13% [5.6%, 26%] |
| Transactional sex | 1.6% [0.41%, 6.3%] | 5.1% [0.93%, 24%] | 4.7% [3.2%, 6.8%] | 7.4% [2.2%, 22%] |
| Child marriage | 0.9% [0.36%, 2.1%] | 0% [0.00%, 0.00%] | 14% [12%, 16%] | 14% [8.1%, 25%] |

SD = Standard Deviation; CI = confidence interval.

^1^ Weighted % (95% CI).

^2^ Completed higher than primary school.

^3^ Participants over 18 years old.

Table S4. Missingness across variables used in the analyses.

| **Variable** | **Missing (%)** |
| --- | --- |
| District | 0.00 |
| Individual weight | 0.00 |
| PSU | 0.00 |
| Age | 0.00 |
| Age group | 0.00 |
| Sex | 0.00 |
| Living with HIV | 0.68 |
| Orphanhood | 0.03 |
| Social protection (Govt.) | 3.11 |
| Social protection (Non-Govt.) | 3.17 |
| Enrolled in school | 2.14 |
| Educational attainment | 0.11 |
| Engaged in any paid work | 2.85 |
| Consistent condom use | 0.56 |
| Multiple sexual partners | 0.62 |
| Transactional sex | 0.27 |
| Child marriage | 0.17 |


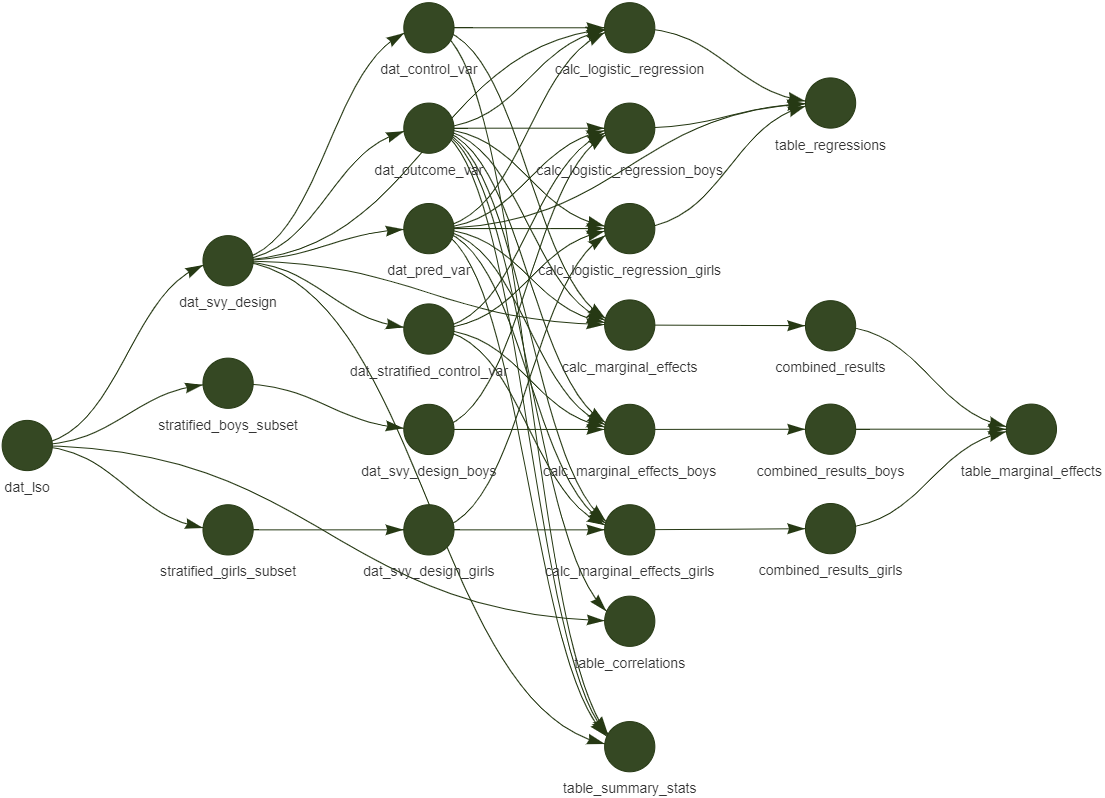


Figure S1. Visual representation of the interactive network graph of the workflow and dependencies between different analysis steps using the package {targets} in R (1).

Green circles represent target objects and contain defined functions for specific analysis steps. For example, dat_lso loads the dataset and makes adjustments to prepare for data analysis, followed by dat_svy_design (which declares the survey design for the whole sample), and stratified_boys_subset and stratified_girls_subset (which selects only males and only females from the entire sample, respectively).


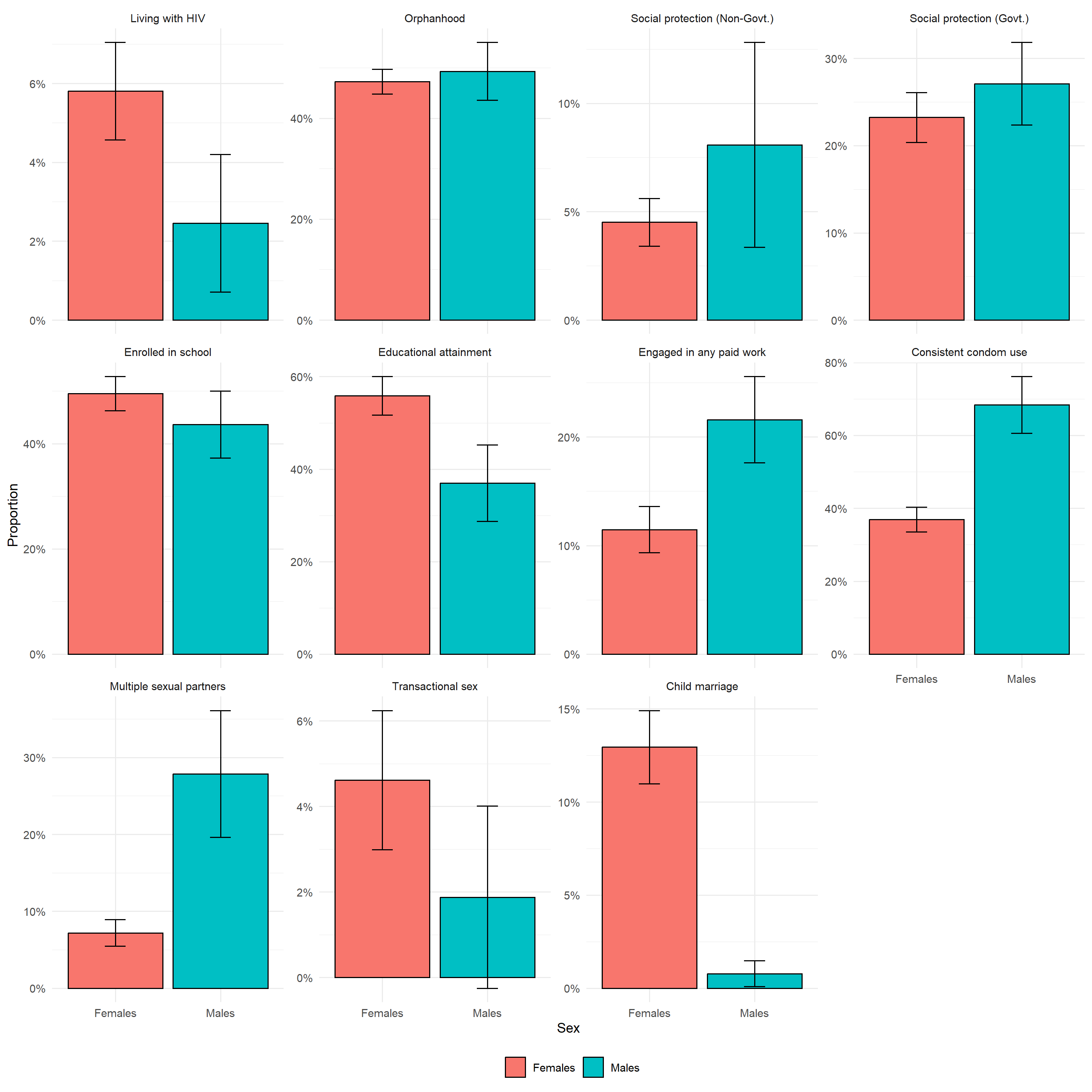


Figure S2. Selected sociodemographic characteristics of 13-24-year-old adolescents and young people living in poverty (lower two wealth quintiles)

*Engagement in any paid work only refers to participants over 18 years old.


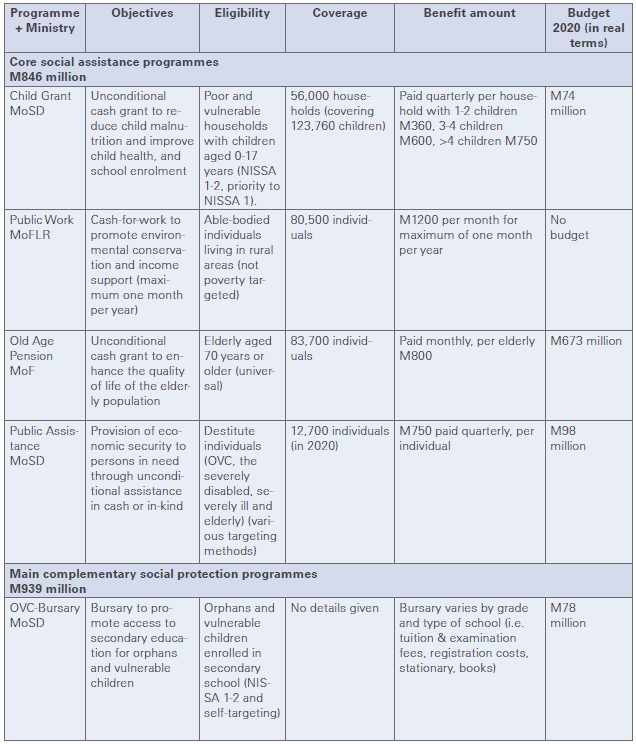


Figure S3. Summary of existing social protection programmes in Lesotho(2).


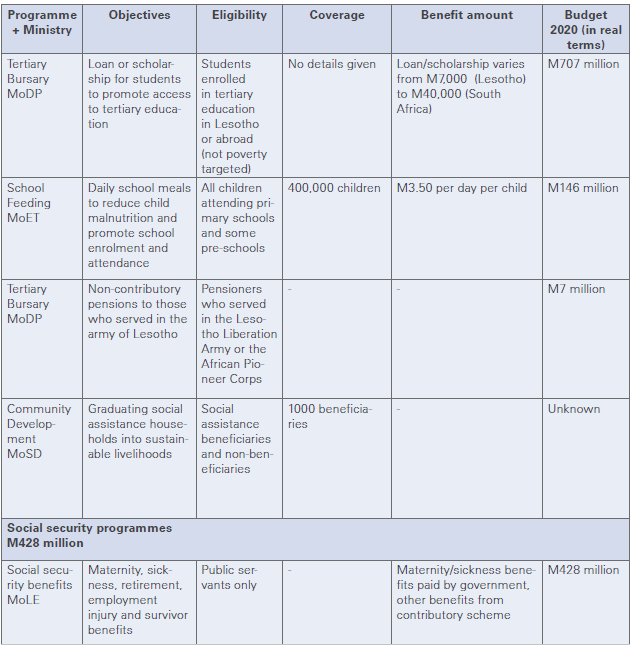


Figure S4. Summary of existing social protection programmes in Lesotho (2).


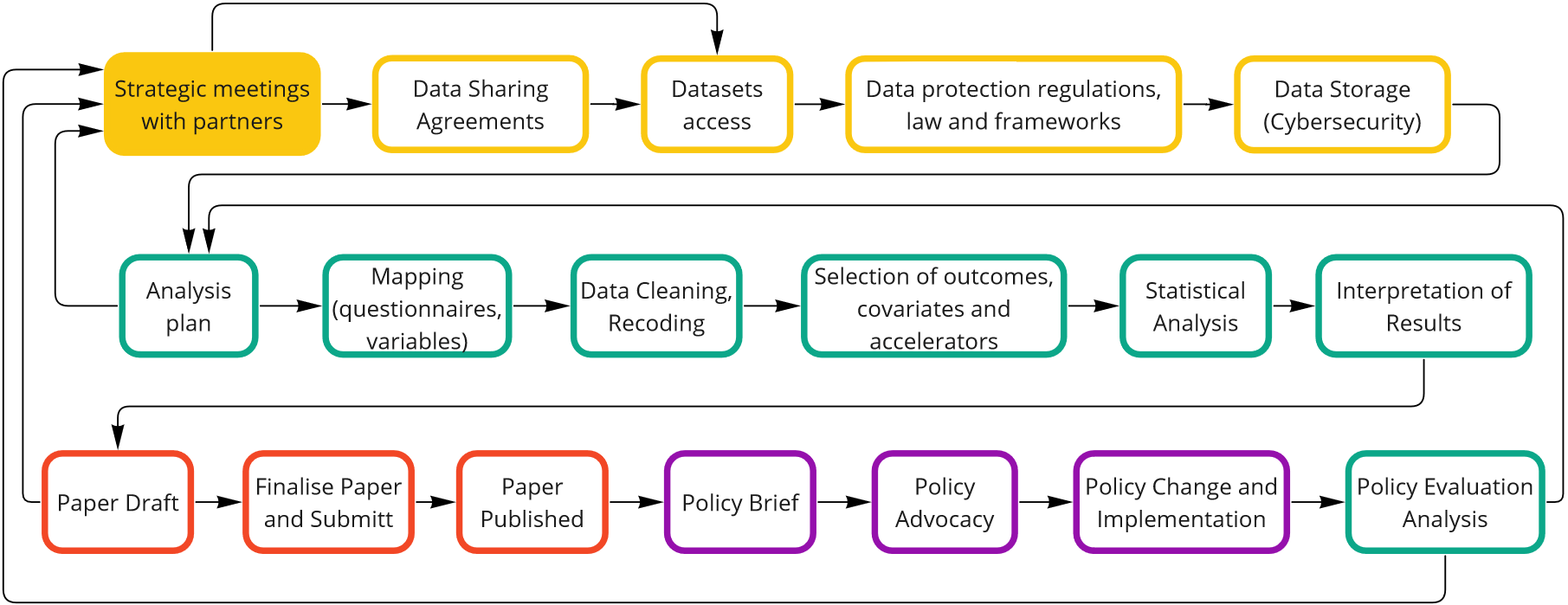


Figure S5. Summary of the secondary data analysis workflow and relationships with data management strategies and policy-driven research projects.

Figure S5 shows the secondary data analysis strategy and workflow for conducting policy-driven research using large survey data with sensitivity information. Iterations focus on the participation of partner institutions that provide continuous feedback on the work. Further details on data management strategies are published elsewhere (3).

References

1. Landau W. The targets R package: a dynamic Make-like function-oriented pipeline toolkit for reproducibility and high-performance computing. JOSS. 2021 Jan 15;6(57):2959.

2. Government of the Kingdom of Lesotho. Lesotho National Social Protection Strategy II: 2021-2031 [Internet]. Maseru; 2022. Available from: https://www.gov.ls/download/lesotho-national-social-protection-strategy-ii/

3. Hertzog L, Chen-Charles J, Wittesaele C, De Graaf K, Titus R, Kelly J, et al. Data management instruments to protect the personal information of children and adolescents in sub-Saharan Africa. IQ [Internet]. 2023 Jun 30 [cited 2023 Jul 17];47(2). Available from: https://iassistquarterly.com/index.php/iassist/article/view/1044
